# Low frequency of Y anomaly detected in Australian Brahman cow-herds

**DOI:** 10.1016/j.mgene.2015.01.001

**Published:** 2015-02-14

**Authors:** Gregório M.F. de Camargo, Laercio R. Porto-Neto, Marina R.S. Fortes, Rowan J. Bunch, Humberto Tonhati, Antonio Reverter, Stephen S. Moore, Sigrid A. Lehnert

**Affiliations:** a Universidade Estadual Paulista (Unesp), Departamento de Zootecnia, Jaboticabal, SP 14884-900, Brazil; b CSIRO Agriculture, Queensland Bioscience Precinct, Brisbane, QLD 4067, Australia; c School of Chemistry and Molecular Biosciences, The University of Queensland, Brisbane, QLD 4067, Australia; d Queensland Alliance for Agriculture and Food Innovation, Centre for Animal Science, The University of Queensland, Brisbane, QLD 4067, Australia

**Keywords:** *Bos indicus*, Reproduction, Sex chromosomes, Fertility, Y translocation

## Abstract

Indicine cattle have lower reproductive performance in comparison to taurine. A chromosomal anomaly characterized by the presence Y markers in females was reported and associated with infertility in cattle. The aim of this study was to investigate the occurrence of the anomaly in Brahman cows. Brahman cows (n = 929) were genotyped for a Y chromosome specific region using real time-PCR. Only six out of 929 cows had the anomaly (0.6%). The anomaly frequency was much lower in Brahman cows than in the crossbred population, in which it was first detected. It also seems that the anomaly doesn't affect pregnancy in the population. Due to the low frequency, association analyses couldn't be executed. Further, SNP signal of the pseudoautosomal boundary region of the Y chromosome was investigated using HD SNP chip. Pooled DNA of “non-pregnant” and “pregnant” cows were compared and no difference in SNP allele frequency was observed. Results suggest that the anomaly had a very low frequency in this Australian Brahman population and had no effect on reproduction. Further studies comparing pregnant cows and cows that failed to conceive should be executed after better assembly and annotation of the Y chromosome in cattle.

## Highlights

• Low frequency of the Y anomaly in Australian cow population.
• The anomaly doesn't affect the pregnancy rates.

Selection for reproductive traits is important for beef cattle production in tropical regions. The impact of reproductive performance on farm productivity may be four to thirteen times more important than growth and carcass traits (Brumatti et al., 2011). Indicine cattle are mostly used in tropical areas and have lower reproductive rates when compared to the taurine subspecies (Lunstra and Cundiff, 2003, Abeygunawardena and Dematawewa, 2004).

McDaneld et al. (2012) described an anomaly related to the Y chromosome in a crossbred population of cows. This anomaly manifested as Y chromosome markers that were detectable in females. The authors reported that cows that carry this anomaly in their studied populations, in the USA, failed to conceive in two subsequent breeding seasons. Our aim was to investigate the existence of the anomaly in two Australian Brahman cattle herds and verify its association with reproductive traits.

First trial: Brahman cows (n = 929) from the Beef CRC population with reproductive phenotypes were studied. The phenotypes were age at puberty, estimated from the observation of the first corpus luteum (929 records), and length of post-partum anoestrus interval (617 records), measured in number of days between birth of the first calf and resumption of ovulation. Detailed information about this population and phenotypes can be found in Hawken et al. (2012).

The GAPDH and BOV_Y primers pairs described by Park et al. (2001) and McDaneld et al. (2012) were used in quantitative real time-PCR assays performed in triplicate for all cows. The GAPDH primers were used as an amplification control and the BOV_Y primers were specific for the Y chromosome. The specific primers indicated the presence of the translocation and the animals were identified as “carriers” or “not carriers”, being impossible to differentiate between the heterozygous (one X chromosome translocated) and homozygous (both X chromosomes translocated) for the group of “carriers”. These 929 cows were individually genotyped. Amplification results with Ct values higher than 30 cycles were discarded.

Only six out of 929 cows in the Australian Brahman population showed amplification of the Y-chromosome fragment (Ct values between 14 and 23 cycles). These animals were considered carriers of the Y anomaly. The frequency of the anomaly was 0.6% in the population. Due to the low frequency, we could not execute association analyses with reproductive phenotypes. McDaneld et al. (2012) reported a frequency of 18% to 29% in non-pregnant/low reproductive populations. Results suggest that the anomaly had low frequency in this Australian Brahman population, in contrast to the US populations studied by McDaneld et al. (2012).

Results presented here indicate no association between the Y anomaly and failure to conceive. From the 929 cows genotyped, 617 conceived at least once. After the first breeding season, 312 non-pregnant cows were genotyped before being excluded from the population. Out of the 6 carriers of the Y anomaly, 5 conceived at least once. Results obtained by McDaneld et al. (2012) imply that cows with the Y anomaly never conceived. However, the same authors indicate that the fragment size of the translocated Y chromosome could vary, altering its impact. The Australian cows could have acquired a smaller translocated fragment of the Y-chromosome that does not affect conception.

Second trial: As to confirm the results of the first trial, pools of blood samples of females that were diagnosed as “non-pregnant” or “pregnant” were genotyped according to the sampling methods of pooled DNA described in (Reverter et al., 2014). For pooled genotyping, blood samples were collected from commercial Brahman heifers at their first pregnancy test and pooled according to their pregnancy status. The “non-pregnant” or “pregnant” pools were from 27 and 29 animals, respectively. There was no relationship between the two phenotypes of cows (pregnant x non-pregnant), apart from them being from the same breed. Within the pools of the same phenotype, the cows could be half-sibs, but all were from the same birth-year, so there are no mother/daughter relationships. Pooled DNA was genotyped using the Illumina bovine 770 K HD bovine chip.

We examined the signal of SNPs from the bovine HD chip that map to the boundary of the pseudoautosomal region (29 Mb-30 Mb) of the Y chromosome. The intensity of the signal was low and similar among pools of pregnant and non-pregnant cows (data not shown).

In conclusion, in the Australian Brahman cattle studied, the Y anomaly was detected at a very low frequency, and did not appear to be incompatible with pregnancy success. Additional examples of females carrying the Y anomaly might be found if populations of females that had two consecutive failed breeding seasons were studied. To study the nature of the Y translocation in more detail, it would be an advantage to access a completed assembly and gene annotation of the Y chromosome in cattle, which is not yet available.

## Competing interests

The authors declare no completing interests.

## Abbreviations

Beef CRC: Cooperative Research Centre for Beef Genetic Technologies
GAPDH: Glyceraldehyde 3-phosphate dehydrogenase
PCR: polymerase chain reaction
HD: high density
SNP: single nucleotide polymorphism
